# Generation of a Novel Regulatory NK Cell Subset from Peripheral Blood CD34^+^ Progenitors Promoted by Membrane-Bound IL-15

**DOI:** 10.1371/journal.pone.0002241

**Published:** 2008-05-21

**Authors:** Massimo Giuliani, Julien Giron-Michel, Simone Negrini, Paola Vacca, Deniz Durali, Anne Caignard, Caroline Le Bousse-Kerdiles, Salem Chouaib, Aurore Devocelle, Rajia Bahri, Antoine Durrbach, Yassine Taoufik, Silvano Ferrini, Michela Croce, Maria Cristina Mingari, Lorenzo Moretta, Bruno Azzarone

**Affiliations:** 1 INSERM, UMR 542, Université de Paris XI, Hôpital Paul Brousse, Villejuif, France; 2 Istituto Giannina Gaslini, Genova, Italy; 3 Dipartimento di Medicina Interna, Università di Genova, Genova, Italy; 4 Centro di Eccelenza per la Ricerca Biomedia, University of Genoa, Genoa, Italy; 5 Istituto Nazonale per la Ricerca sul Cancro, Genoa, Italy; 6 INSERM UMR 802, Université de Paris XI, Le Kremlin-Bicêtre, France; 7 INSERM UMR 753, Université de Paris XI, Institut Gustave Roussy (IGR), Villejuif, France; 8 INSERM UMR 602, Université de Paris XI, Hôpital Paul Brousse, Villejuif, France; 9 Laboratory of Immunotherapy, Istituto Nazionale per la Ricerca sul Cancro, Genova, Italy; Oklahoma Medical Research Foundation, United States of America

## Abstract

**Background:**

NK cells have been long time considered as cytotoxic lymphocytes competent in killing virus-infected cells and tumors. However, NK cells may also play essential immuno-regulatory functions. In this context, the real existence of a defined NK subset with negative regulatory properties has been hypothesized but never clearly demonstrated.

**Methodology/Principal Findings:**

Herein, we show the *in vitro* generation from human peripheral blood haematopoietic progenitors (PB-HP), of a novel subset of non-cytolytic NK cells displaying a mature phenotype and remarkable immuno-regulatory functions (NK-ireg). The main functional hallmark of these NK-ireg cells is represented by the surface expression/release of HLA-G, a major immunosuppressive molecule. In addition, NK-ireg cells secrete two powerful immuno-regulatory factors: IL-10 and IL-21. Through these factors, NK-ireg cells act as effectors of the down-regulation of the immune response: reconverting mature myeloid DC (mDC) into immature/tolerogenic DC, blocking cytolytic functions on conventional NK cells and inducing HLA-G membrane expression on PB-derived monocytes. The generation of “NK-ireg” cells is obtained, by default, in culture conditions favouring cell-to-cell contacts, and it is strictly dependent on reciprocal trans-presentation of membrane-bound IL-15 forms constitutively and selectively expressed by human CD34^+^ PB-HP. Finally, a small subset of NKp46^+^ HLA-G^+^ IL-10^+^ is detected within freshly isolated decidual NK cells, suggesting that these cells could represent an *in vivo* counterpart of the NK-ireg cells.

**Conclusions/Significance:**

In conclusion, NK-ireg cells represent a novel truly differentiated non-cytolytic NK subset with a self-sustainable phenotype (CD56^+^ CD16^+^ NKp30^+^ NKp44^+^ NKp46^+^ CD94^+^ CD69^+^ CCR7^+^) generated from specific pSTAT6^+^ GATA3^+^ precursors. NK-ireg cells could be employed to develop new immuno-suppressive strategies in autoimmune diseases, transplant rejection or graft versus host diseases. In addition, NK-ireg cells can be easily derived from peripheral blood of the patients and could constitute an autologous biotherapic tool to be used combined or in alternative to other immuno-regulatory cells.

## Introduction

Natural Killer (NK) cells, traditionally considered to be major innate effector cells, have been long time relegated to the job of killing virus-infected cells and tumors. However, more and more evidence has been obtained that NK cells play positive or negative regulatory effect by secreting various cytokines or cell-to-cell contact and maintain immune homeostasis [1]–[5]. As a consequence, subsets of NK cells in mouse and human have been defined based on their cell-surface phenotype and functional properties that are shaped by environmental factors among which cytokines play a major role [1]–[5]. In this context, the differentiation of CD34^+^ HP into NK cells is strictly dependent on Interleukin 15 (IL-15) [6], [7]. However, the mechanism by which IL-15 mediates NK cell differentiation is not fully understood and it seems to be only partially dependent on the soluble form of this cytokine.

Although soluble IL-15 can bind to the IL-15R complex and induce signals in a manner similar to other cytokines upon interaction with their receptors [8], [9], there is increasing evidence that IL-15 mediates many specific biological responses in cell membrane-associated forms [10]–[12]. In this context, membrane-bound IL-15 (mb-IL-15) anchored through the IL-15Rα chain on the surface of Bone Marrow (BM) myeloid accessory cells, in the mouse model, is essential for normal development of NK cells [13], [14], whereas IL-15 *trans*-presentation seems to be more complex in human as suggested by the detection of additional forms of mb-IL-15 independent on the IL-15Rα [10], [12].

Indeed, human spleen myofibroblasts display a mb-IL-15 associated with IL-15Rβγc chains [12], which is necessary and sufficient to trigger *in vitro* the differentiation of circulating progenitors into cytolytic NK cell [15]. Finally, prostate cancer cells express a trans- membrane IL-15, anchored in a IL-15R-independent fashion, which can participate in reverse signaling and/or act in a juxtacrine fashion promoting the development of bystander cells [10]. These data indicate that IL-15 trans-presentation by accessory cells seems to be an important requirement for NK cell homeostasis [13]-[15], while it is still poorly defined whether IL-15 trans-presented by CD34^+^ HP may play a role in this process.

In this respect, we have recently observed that human uncommitted CD34^+^ PB-HP constitutively express mb-IL-15 [16], the involvement of which in the commitment towards the NK differentiation pathway has not been explored. In order to clarify this point, we expanded human CD34^+^ PB-HP adapting previously described protocols, based on the use of media supplemented with stem cell factor (SCF) and flt3 ligand (FL), that induce an enrichment in CD34^+^ CD56^−^ NK progenitors (NKP) [17], [18]. Surprisingly, the results revealed that membrane-bound IL-15 forms present on human PB-HP [10], [12], in conditions favouring cell-to-cell contact, promote, through reciprocal trans-presentation, the generation of a novel subset of mature non-cytolytic NK cells. These NK cells secrete major regulatory factors (IL-10, IL-21 and HLA-G) that likely mediate tolerogenic/immunosuppressive activities on myeloid DC maturation, on NK associated cytolytic functions and on PB monocytes phenotype and function.

The real existence of a NK subset with negative regulatory functions has been hypothesized but never clearly demonstrated [2], [5]. Herein, we show the mb-IL-15 dependent generation from human PB-HP, of a novel subset of mature non–cytolytic NK cells (NK-ireg), whose main functional hallmark is the surface expression/release of a major immunosuppressive molecule HLA-G [19], and that display remarkable regulatory functions.

## Materials and Methods

### Cytokines and reagents

Human recombinant IL-4, soluble IL-15Rα/Fc Chimera (s-IL-15Rα), soluble IL-21R/Fc Chimera (s-IL-21R), the Cytofix/Cytoperm reagent are from R&D Systems (Lille, France). Recombinant Stem Cell Factor (SCF), IL-12, IL-21, GM-CSF and FMS-like tyrosine kinase 3 ligand (Flt3-L) were purchased from Immunotools (Friesoythe, Germany). LPS (*E. Coli,* 055:B5), Brefaldin A, PHA, and PMA were from Sigma-Aldrich (S. Q. Fallavier France). Antibodies used in this study are listed in table 1.

**Table 1:** Antibodies, origin and use

Abs anti-	origin	use
IL-15Rα (AF 247)	R&D Systems (Lille, France).	FLC+Neu
IL-10-PE, IL-15-PE, IFNγ-FITC		FLC
IL-12		FLC
IL-21	eBioscience (San Diego USA)	W.B.
IL-15Rβ (6E8)	Y. Jacques (U463 INSERM, Nantes, France)	Neu
IL-2/IL-15-γc (TUGh4),	BD PharMingen (Le Pont de Claix, France).	Neu
CD107a, CD85j		FLC
CD11c-PE, CD16-PE, CD34-PE, CD38-PE, CD56-PE, CD69-PE, HLA-DR-FITC	Immunotools (Friesoythe, Germany)	FLC
Perforin-FITC, Granzyme B-FITC		FLC CF
CD80-PE, CD83-PE, CD86-PE, NKp30-PE, NKp44-PE, NKp46-PE, NKG2D-PE, CD94-PE ,CD161-PE	Beckman Coulter (Villepinte, France)	FLC
isotype matched Ig controls		FLC
CCR7	BD Biosciences (San Diego, United States)	FLC
HLA-G (4H84), TGF-β1 pSTAT3, pSTAT6, GATA3	Santa-Cruz Biotechnologies (Tebu, Le Parray en Yvelines, France)	FLC+WB W.B
PE-or FITC-labeled goat anti-mouse and goat anti-rabbit	Jackson ImmunoResearch Laboratories, France)	FLC secondary Abs.
Alexa Fluor488-labelled goat anti-rabbit and goat anti-mouse	Molecular Probes, Interchim, France	FLC CF secondary Abs.
horseradish peroxidase (HRP) labelled goat anti mouse	Amersham Biosciences, Orsay, France	W.B. secondary Abs.
HLA-G (MEMG/9-PE, 87G azide free, 0IG-FITC)	ExBio , Prague,	FLC,Neu, CM

FLC = flow cytometry, W.B. = Western Blot, CF = Confocal Microscopy, Neu. = neutralization

### Cell Lines

The acute T cell lymphoblastic leukaemia Jurkat, the erythro-leukemic cell line K562, the melanoma cell line FON, the promyelocytic leukemia cell line HL-60 and the neuroblastoma Neuro2a were cultured in RPM1 1640 medium (GIBCO, Eragny, France) supplemented with 2 mM L-glutamine, HEPES, 1% of penicillin streptomycin solution (GIBCO), 10% heat-inactivated fetal calf serum (PAA Laboratories Les Mureaux, France).

### Purification of CD34^+^ PB-HP and commitment to the NK pathway

CD34^+^ PB-HP were purified (>90%) from fresh PBMC of healthy donors as previously described [20] according to standard procedures by a direct immunomagnetic method (Milteneyi Biotech, Paris, France). Then, PB-HP were committed to NK pathway expanding the cells in STEMα−Α medium (Stem Alpha, Saint Clement les places, France) supplemented with SCF and FL (both at 100 ng/ml), a condition that favours and enriches the frequency of NK progenitors [18]. PB-HP were seeded at 5×10^5^cells/ml, in order to magnify cell-cell interactions.

### Isolation of d-NK and PB-derived NK cells

Samples were obtained at 9 to 12 weeks of gestation from singleton pregnancies of mothers requesting termination of the pregnancy for social reasons or who were undergoing evacuation of retained products of conception following spontaneous pregnancy failure. The study was approved by the relevant institutional review boards and all patients gave their written informed consent according to the Declaration of Helsinki. Decidual tissue was separated from specimens obtained by suction evacuation of the uterus. The total decidual tissue was then minced into fragments of 1 mm^3^ and digested for 1 hour at room temperature under agitation in PBS with 200 U/mL hyaluronidase (Sigma) and 1 mg/mL collagenase type IV (Seromed, Berlin, Germany). The cell suspension was filtered through sterile stainless-steel 100-µm wire mesh and washed once in PBS. The mononuclear cell population was isolated using Ficoll-Hypaque density gradient (Sigma), washed twice in PBS, and used for cell isolation or fluorescence analysis. To obtain enriched NK cells, both decidual mononuclear cells and PBMC were depleted of CD4^+^, CD14^+^, CD19^+^, CD3^+^,Vδ1^+^, and Vδ2^+^ cells by negative selection using appropriate mAbs followed by goat antimouse-coated Dynabeads (Dynal, Oslo, Norway) and immunomagnetic depletion. Peripheral-blood lymphocytes were isolated from peripheral blood from healthy donors and pregnant woman using Ficoll-Hypaque density gradient either directly or after enrichment (>95%) for NK cells using Rosettesep (StemCell Technologies, Meylan, France). PB-derived NK cells were used as freshly isolated resting cells or activated with 100 U/ml of IL-2 for 4 days.

### Generation of PB-derived human allogeneic myeloid DC and PB-NKP/DC co-culture

Monocytes were purified from PBMC (>95%) by using the CD14-Adembeads (Ademtech, Pessac, France) and DC were generated by culturing monocytes in RPMI 1640 complete medium supplemented with GM-CSF and IL-4 (both at 50 ng/ml). On day 6, immature DC were incubated with LPS (1 µg/ml) for 48 h to generate myeloid mature DC (mDC). NK/DC cross-talk was studied co-culturing for 24–48 h mDC with allogenic 3 weeks-old PB-NKP, or using PB-NKP cell supernatants. DC morphology was assessed on cytospin preparations after RAL 555 staining Kit (Abcells, Tampere France). Pictures were obtained with an Olympus microscope (Arcueil, France) equipped with a 40x objective lens using a CoolPix 995 numeric camera (Nikon Paris, France).

### Cytotoxicity assay

IL-2 activated NK cells and 3 weeks-old PB-NKP were tested for cytolytic activity in a standard 4-h ^51^Cr release assay as previously described [21] in the presence or absence of 5 ng/ml of IL-12. K562 human leukemic cells were used as target in experiments of natural cytotoxicity. The effector-to-target (E/T) ratios were 10∶1.

### Mixed lymphocyte reaction (MLR)

Allogeneic PBMC were labelled with 1 µM of 5,6-carboxyfluorescein diacetate-succinimyl ester (CFSE) in PBS/0.1% BSA for 10 min at 37°C and washed with complete medium. Cells were then seeded in 96-well flat bottom microtiter plates (Costar) at 1×10^6^/ml in complete culture medium with mitomycin treated mDC (DC/PBMC ratio 1/10) pre-incubated or not for 24 h with PB-NKP (mDC/PB-NKP ratio 1/1). Proliferation was analyzed by flow cytometry after 5 days.

### Cell Phenotype and cytokines detection by flow cytometry

Cell surface antigen expression was evaluated on suspensions of living cells by cytofluorimetry analysis as previously described [20]. Since IL-10, TGFβ and IFNγ may be detected as surface-bound cytokines [21]–[23], we exploited this property investigating the production of these cytokines by flow cytometry after rapid direct staining (30 min at 4°C) of a suspension of living cells. Cell surface cytokine expression was validated by further intracellular detection using the Cytofix/Cytoperm reagent according to the manufacturer's instructions. Flow cytometric analysis was performed using FACScalibur (Becton Dickinson, Saint Quentin Yvelines, France), using CellQuest (BD Biosciences) and WinMDI (Scripps Research Institute, La Jolla, CA) software programs. At least 5000 events were analyzed in each test.

### Confocal laser-scanning microscopy

Expression of granzyme B or perforin and nuclear localization of phospho-STAT6 and GATA3 were analyzed by confocal microscopy on PB-NKP pre-treated or not with s-IL-15Rα for 1h. Nuclear localization of phospho-STAT3 was analyzed in LPS-activated mDC co-cultured overnight or not with PB-NKP. Cell permeabilization, intracellular staining and processing for confocal microscopy were performed as previously described [20]. The slides were examined by confocal laser microscopy (Leica TCS Confocal System, Wetzler, Germany).

### Western Blotting

PB-NKP and control cell lines were used: Jurkat cells stimulated for 30 min with IL-4 at 10 ng/ml for pSTAT-6 and GATA3, HL-60 cell line for IL-21 and FON melanoma cell line for HLA-G detection. PBL and IL-2-stimulated NK cells were used as negative controls. Total cell lysates and pre-filtered, 10-fold concentrated cell supernanants were subsequently processed for Western Blot analysis as previously described [20]. Immunodetection of the protein blotted was determined using a Fujifilm Intelligent Dark Box II. β-actin was used as internal control.

### RT-PCR analysis for human IL-10, IL-21

Total RNA was isolated using the NucleoSpin RNA II kit (Macherey-Nagel, Duren, Germany) according to the manufacturer's instructions. One µg of total RNA was then reverse-transcribed using the SuperScript II Reverse transcriptase (Invitrogen, Milano, Italy) in a final volume of 20 µl. Two µl of the cDNA were separately amplified, in a final volume of 25 µl, with 2.5 IU Taq polymerase (Genecraft, Germany), in the presence of 1 µM of the primers specific for IL-10, IL-21, and for the housekeeping gene β-actin. The following human primers were used: **IL-10,** forward: 5′-GCTCTGTTGCCTGGTCCTCC, reverse : 5′-CTCCACGGCCTTGCTCTTGT (58°C); **IL-21,** forward : 5′-GAAGTGAAAACGAGACCAAGGT, reverse: 5′-CTGCAAGTTAGATCCTCAGG (52°C); **β-actin,** forward: 5′-GGCATCGTGATGGACTCCG reverse: 5′-GCTGGAAGGTGGACAGCGA (61°C). The amplifications were carried out in a PCR Sprint thermal cycler (Hybaid, Ashford, UK). Ten µl of PCR products were then analysed on 1–2% agarose gel stained with ethidium bromide.

### IL-10 and IL-15 enzyme-linked immunosorbent assay (ELISA)

Culture cell supernatants were harvested at different times of culture and tested in triplicate for IL-10 and IL-15 production by ELISA (Immunotools, Friesoythe, Germany and R&D systems: Lille, France) according to the manufacturer's instructions. The ELISA plate was read at OD 405 nm on a Microplate ELISA reader (Titertek multiskan plus, Puteaux, France).

### Data Analysis

All experiments were performed in at least three independent assays, which yielded highly comparable results. Data are summarized as means+/−S.D. Approval was obtained from the INSERM institutional review board for these studies. Informed consent was provided according to the Declaration of Helsinki.

## Results

### Detection of membrane-bound IL-15 on proliferating human Peripheral Blood CD34^+^ progenitors

**Figure 1:**
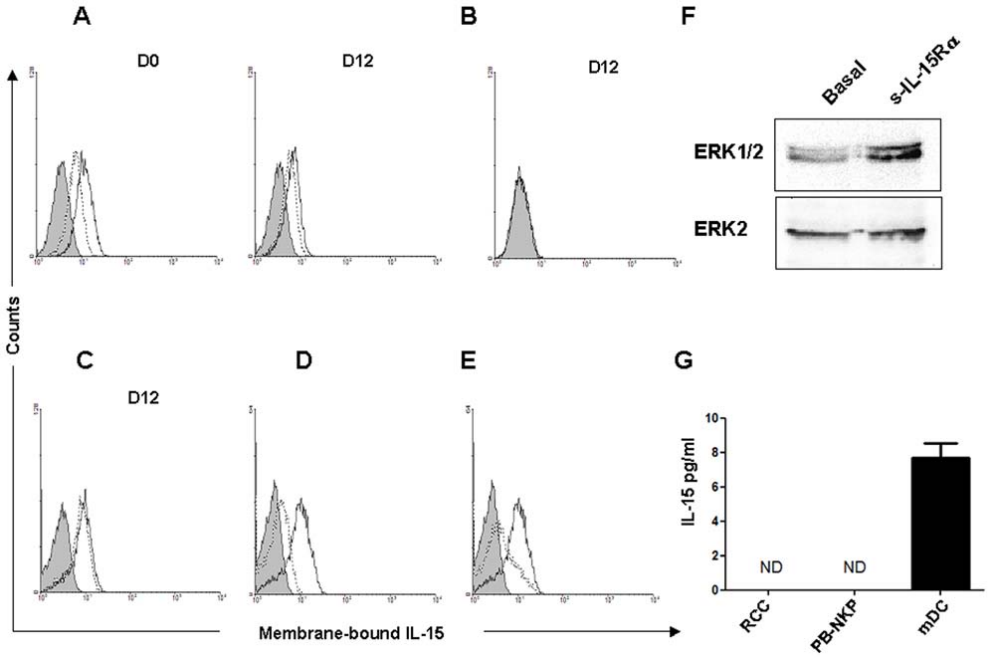
Membrane-bound IL-15 detection on proliferating human circulating CD34^+^ HP. CD34^+^ PB- and BM-HP were committed to the NK cell lineage, culturing in STEMα Α medium supplemented with 100 ng/ml of FL and KL. The presence of membrane-bound IL-15 was evaluated staining the cells with anti-IL-15 mAb 247-PE (continuous line) or isotype-matched control Abs (shadowed peaks), followed by FACS analysis. (A) Constitutive expression and effect of the treatment at 37°c with 10 µg/ml of neutralizing mAbs directed against the IL-15Rα subunits (dotted lines). (B) No mb-IL-15 expression on BM-NKP. (C) PB-NKP cells were also treated with acidic buffer (pH 3.5; dotted lines) which does not modify their mb-IL-15 expression. (D, E) In contrast, acidic shock and treatment with neutralizing anti-IL-15Rα mAbs cause the suppression of mb-IL-15 form expressed by the erythroleukemic cell line TF1. (F) Induction of mb-IL-15-dependent reverse signal. Western Blot analysis of MAPKinase ERK1/2 activation after 15 minutes treatment of PB-NKP with the soluble recombinant IL-15Rα. (G) ELISA assay shows that PB-NKP do not secrete IL-15. RCC and mDC supernatants were used as negative and positive controls of secretion respectively.

Quiescent human haematopoietic progenitors constitutively secrete IL-15 that plays a role in their homeostasis but is not competent for sustaining their autonomous commitment to the NK cell pathway [20], [24]. We investigated the form and function of IL-15 expressed by CD34^+^ HP cultured under conditions (STEMα Α medium supplemented with 100 ng/ml of SCF and FL) that were shown to induce an enrichment in CD34^+^ CD56^−^ NK cell progenitors (NKP) [17], [18]. In figure 1, flow cytometric analysis shows that PB-HP (Fig. 1A), but not BM- derived ones (Fig. 1B) express a mb-IL-15. This form is detected on freshly isolated PB-HP (Fig. 1A, D0) and it is maintained during the following days in culture (Fig. 1A, D12). The treatment of these cells with neutralizing mAbs directed against the IL-15Rα subunit (10 µg/ml) only partially inhibited (20%) the detection of mb-IL-15 (Fig. 1A). The consistent finding of residual expression of mb-IL-15, not inhibited by anti-IL-15Rα mAbs suggested the presence of an IL-15 form anchored in an IL-15R-independent fashion. While IL-15 noncovalently bound to the cell surface via its interaction with IL-15Rα is released by acidic treatment [11], IL-15 anchored to the cell membrane is not [10]. We observed that acidic treatment does not reduce mb-IL-15 on PB-HP (Fig. 1C, D12), while in the erythroleukemic cell line TF1, the treatment with neutralizing mAbs against IL-15Rα subunit or the acidic shock causes the disappearance of the mb-IL-15 anchored through the IL-15Rα (Fig. 1D and 1E). In addition, treatment of PB-HP with the soluble IL-15Rα induces the phosphorylation of the MAPkinase ERK 1/2 (Fig. 1F), indicating that this treatment activates, as recently reported in cells expressing a trans-membrane IL-15 form, a reverse signal.

Altogether the residual expression of mb-IL-15 following treatment with anti-IL-15R mAbs, its resistance to acidic treatment and the activation of a reverse signal in response to the soluble receptor suggest the existence of an IL-15 form anchored in an IL-15R independent fashion that could correspond to the trans-membrane IL-15 form recently described on human prostate cancer cells [10]. Finally, IL-15 ELISA assay on the supernatants from cultured PB-HP, show that there no detectable IL-15 secretion. Mature myeloid dendritic cells [25] and renal cancer cells [26] were employed as positive and negative controls of IL-15 secretion (Fig. 1G)**.** These results suggest that, soon after immunopurification, two parameters could differentiate PB-HP from BM-HP: the expression of mb-IL-15 and absence of IL-15 secretion.

### NK commitment of PB-HP in the absence of exogenous lymphokines

**Figure 2:**
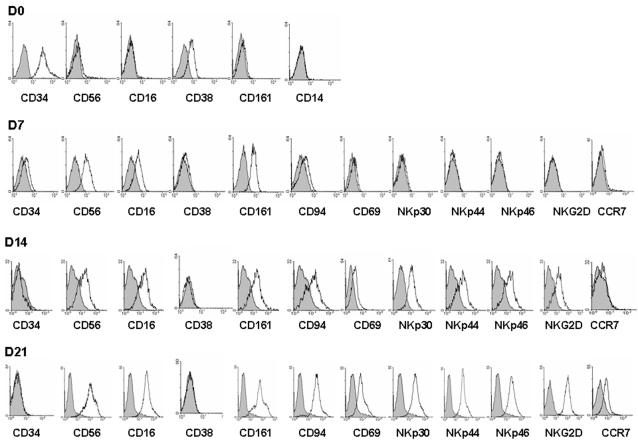
Autonomous NK commitment of PB-HP. Commitment into the NK pathway of PB-HP was evaluated by flow cytometry at different times in culture, analyzing CD34, CD38 and the NK markers CD56, CD16, CD161, CD94, NKp30, NKp44, NKp46, NKG2D, CD69 and CCR7. Shadowed peaks represent staining with isotype control Abs. Continuous lines represent staining with the indicated Abs. The frequency of PB-HP represents about 0.1% of mononuclear cells of the peripheral blood. The data are representative of more than 30 separate experiments.

We next investigated whether the human CD34^+^ PB-HP expressing mb-IL-15 could *trans-*present IL-15 each other mimicking the *in vivo* effect of BM myeloid accessory cells on bystander BM-HP and trigger their commitment towards the NK cell lineage in the absence of exogenous lymphokines. Freshly isolated PB-HP expresses the CD34^+^ CD38^+^ CD56^dull^ CD16^−^ CD161^−^ CD14^−^ surface phenotype (Fig. 2, D0). When these cells are maintained at high cell density that favors cell to cell contact (5×10^5^ cells/ml), there was a progressive expansion of uni-lineage homogeneous committed progenitors that, growing in suspension, progressively acquired the phenotype of mature NK cells. On day 7, these progenitors acquired markers typical of immature NK cells. However, they still maintained low expression of CD34 (CD34^low^ CD56^+^ CD16^low^ CD161^+^ CD94^−/+^) (Fig. 2, D7). At this stage, we frequently observed that a small subset of CD1a adherent cells stems from the cells growing in suspension. This small subset represents less then 3% of the total cell population, but could somehow influence the maturation process of these PB-NKP [16].

After 2 weeks in culture, cells lost CD34 expression and acquired additional NK cell markers (CD69^+^ CD94^+^ NKG2D^+^ NKp30^+^NKp44^+^ NKp46^+^) (Fig. 2, D14). After three weeks, the totality of cultured cells displayed the phenotype of mature NK cells (CD56^+^ CD16^+^ CD161^+^ CD69^+^ CD94^+^ NKG2D^+^ NKp30^+^ NKp44^+^ NKp46^+^ CCR7^+^) (Fig. 2, D21).

### Morphology and cytolytic potential of NK cells generated by PB-NKP

**Figure 3:**
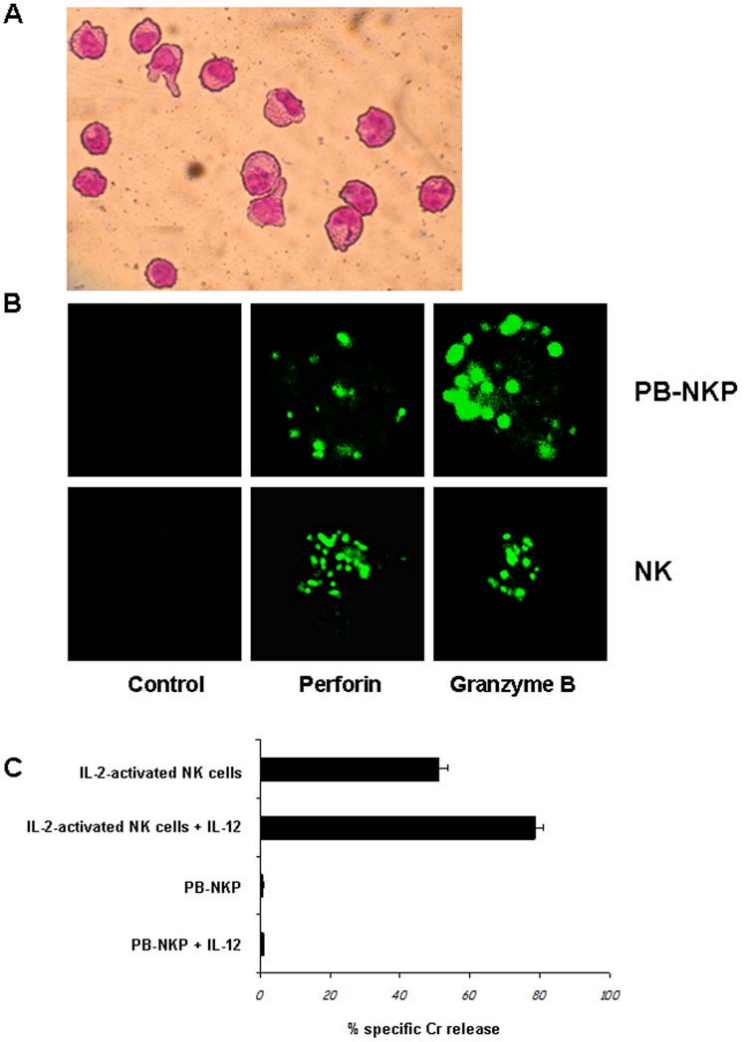
Autonomous NK commitment of PB-HP: morphology and expression of cytolytic granules. (A) Ral 555 staining on 3-weeks-old PB-HP cultured in in STEMα Α medium supplemented with 100 ng/ml of FL and SCF. PB-NKP exhibit a lymphoid morphology. (B) Intracytoplasmic expression of perforin and granzyme B in PB-NKP and in PB activated conventional NK cells was analyzed by confocal microscopy. Both cell types were fixed, permeabilized, and stained for perforin and granzyme B. As negative controls, cells were incubated with mouse IgG, and the second reagent. (C) Analysis of the cytolytic activity of freshly purified NK cells from healthy donors (open boxes) and from PB-NKP (grey boxes) against ^51^Cr-labelled K562 cells. NK cell effectors, treated or not for 18 hours with IL-12 (5 ng/ml), were assayed in a 4-h ^51^Cr-release assay against K562 cells in a dilution of E/T cell ratios (10∶1) in duplicate wells. Data are representative of three experiments performed.

Phenotypical characterization was strengthened by morphological analysis showing that after three weeks in culture PB-HP autonomously committed into the NK pathway display a typical lymphoid morphology (Fig. 3A). In addition, confocal microcopy demonstrated a spotted intracytoplasmic expression of perforin and granzyme B, suggesting their localization within granule-like structures as observed in conventional activated NK cells (Fig. 3B and 3C).

We next compared PB-NKP with IL-2 activated conventional NK cells for their cytolytic potential. Both types of NK cells, treated or not with 5 ng/ml of IL-12 a powerful promoter of NK cells cytolytic activity and functional maturation, were challenged in a 4 hours ^51^Cr-release assay against K562 cells (Fig. 3D). In these experiments, conventional IL-2 stimulated NK cells, display cytolytic activity (50%) which is significantly increased by IL-12 treatment (70%). In contrast, PB-NKP cells do not exhibit any cytolytic potential even at the 10∶1 ratio and in the presence of IL-12. Similar results were obtained using IL-15 or IL-18 alone or associated to IL-12 (data not shown).

### Pattern of activation of transcription factors during NK cell commitment of PB-derived HP

**Figure 4:**
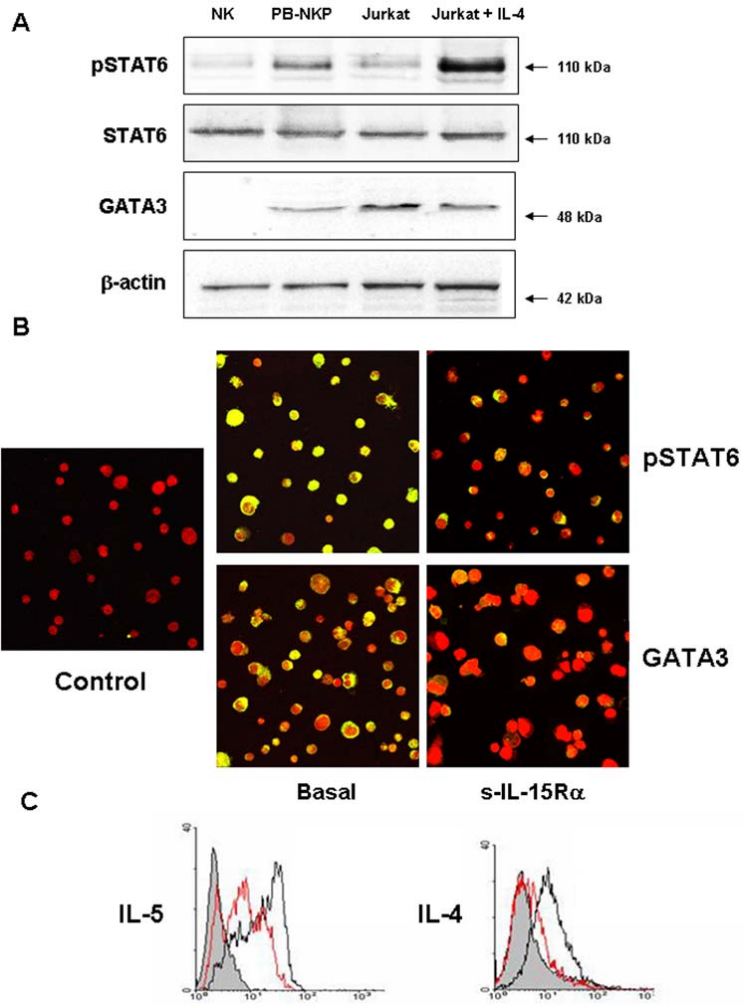
Autonomous NK commitment of PB-HP: expression of Th2 transcription factors. (A) Western blotting detects a specific 110 kDa band for pSTAT6 and a specific 48 kDa for GATA3 in the cell total lysate of PB-NKP but not of conventional NK cells. GATA3^+^ Jurkat cells stimulated with IL-4 were used as controls. (B Left panels) The expression of activated STAT6 and GATA3 was further confirmed, checking by confocal microscopy the nuclear localization of phospho-STAT6 and GATA3 (yellow staining) in PB-NKP. These cells were fixed, permeabilized, and stained for phopho-STAT6 or GATA3 transcription factors (green). Nuclei were stained by propidium iodide (red). As negative controls, cells were incubated with rabbit IgG, the second reagent, and propidium iodide. (B Right panels) Effects of 24 hours pre-incubation of PB-NKP with the soluble IL-15Rα chain (100 ng/ml for 60 min.) on the nuclear localization of phopho-STAT6 or GATA3. (C) Effects of 24 hours pre-incubation with the soluble IL-15Rα chain on the intracellular production of the Th2 cytokines IL-5 and IL-4 in PB-NKP. Intracellular expression of cytokines (continuous black lines) is blocked adding the soluble IL-15Rα chain (continuous red line) for 24 hours. Isotype controls (shadowed peaks).

Subsequently, we analyzed the transcription factor profile expressed by PB-NKP at different culture intervals. We found that, differently from what previously described in Cord Blood NKP (CB-NKP) [12], PB-NKP were characterized by an early and stable activation of STAT6 and the expression of GATA3, two transcription factors that play a key role in the induction of Th2 responses [27], [28]. Indeed, Western Blot analysis revealed that the specific bands of phospho-STAT6 (pSTAT6, 110 kDa) and of GATA3 (48 kDa) were detected in the total lysates of these progenitors, but not of freshly isolated NK cells. GATA3^+^ Jurkat cells stimulated with IL-4 were used as positive controls for pSTAT6 detection [29] (Fig. 4A).

Moreover, the constitutive expression of activated STAT6 and GATA3 in PB-NKP was further confirmed by confocal microscopy showing, that a large majority of the cells displayed nuclear localization of pSTAT6 and GATA3 (Fig. 4B left panels, nuclear yellow staining). Analysis performed on different donors showed that the constitutive activation of STAT6 and GATA3 is a constant and specific feature of these PB-NKP_pSTAT6+GATA3+_ (NKP_SG_).

Since exposure of the mb-IL-15 present on PB-HP to the soluble IL-15Rα/Fc Chimera (s-IL-15Rα) induces a reverse signal activating the MAPkinase ERK (Fig. 1F), we investigated if other signaling pathways were modified after incubation of PB-NKP with the soluble receptor. Confocal microscopy shows that treatment with the s-IL-15Rα inhibits within one hour the nuclear translocation of the two Th2 transcription factors pSTAT6 and GATA 3 (Fig. 4B right panels). In addition, flow cytometry demonstrates that PB-NKP produce Th2 cytokines as shown by the important intracellular storage of IL-5 and IL-4. Intracellular detection of both Th2 cytokines is inhibited after 24 hours exposure to the s-IL-15Rα chain (Fig. 4C). These data show that, in PB-NKP, the constitutive activation of Th2 transcription factors and production of Th2 cytokines is controlled by the endogenous mb-IL-15.

### NK cell commitment of PB-HP is controlled by endogenous mb-IL-15

**Figure 5:**
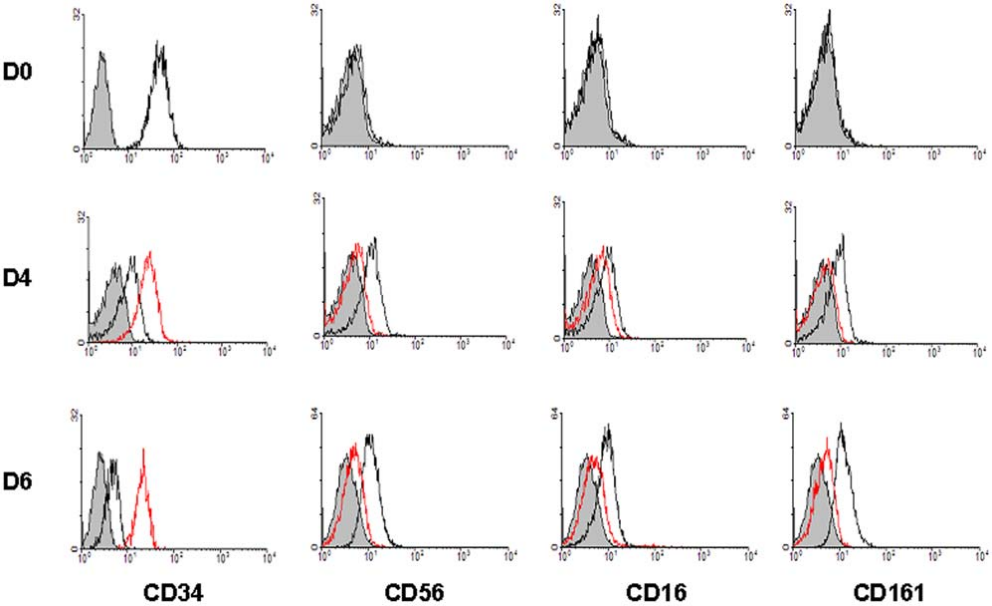
Interference on mb-IL-15 functions causes the down-regulation of NK markers. To test the hypothesis that PB-HP could be committed into the NK pathway under the control of the endogenous mb-IL-15, IL-15 *trans*-presentation was blocked adding neutralizing IL-15Rα (continuous red line) mAbs at the first and at the fourth day of the cell culture. Blocking IL-15Rα chain causes the inhibition of CD56, CD16 and CD161 expression (D4-D6), while the treatment preserves the initial level of CD34 expression (D4-D6). NK markers expression (continuous black lines). Isotype controls (shadowed peaks).

The constitutive expression of mb-IL-15 and the finding that PB-HP could evolve rapidly towards the NK cell pathway suggested that the differentiation of PB-HP into NKP_SG_ may be under the control of endogenous mb-IL-15. In order to test this hypothesis, we attempted to interfere with the mb-IL-15 functions by the use of neutralizing mAbs recognizing the different IL-15R subunits in order to interfere with the juxtacrine loop activated through IL-15 *trans*-presentation. Early addition of IL-15Rα neutralizing mAbs to PB-HP resulted, within four days, in inhibition of CD56 while CD34 surface expression was preserved (Fig. 5, D4). The same treatment was repeated after four days in culture and PB-HP were analyzed 48 hours later. Addition of neutralizing IL-15Rα mAbs (AF247) resulted in down-regulation of the expression of CD56, CD16 and CD161, while the initial expression of the CD34 molecule was maintained (Fig. 5, D6). In contrast, the use of anti-IL-15Rβγc neutralizing mAbs did not modify the surface expression of NK markers (data not shown) suggesting a direct role for the IL-15Rα chain in receiving the signal of *trans*-presented IL-15 for NK commitment.

### NKP_SG_ cells display regulatory functions on the maturation of myeloid dendritic cells

**Figure 6:**
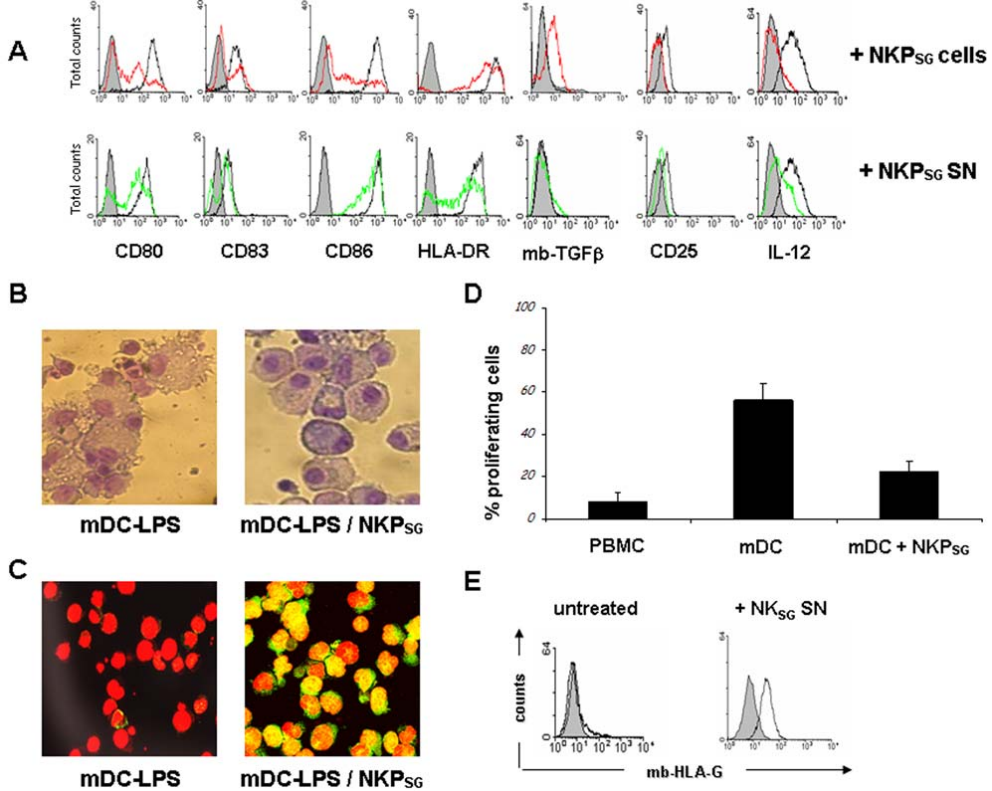
NKP_SG_ cells display regulatory functions on the maturation of myeloid dendritic cells and PB-derived monocytes. (A) Flow cytometry show the modifications of the phenotype of mature myeloid dendritic cells (mDC, black lines), after co-culture of 3 weeks-old NKP_SG_ cells (continuous red lines) or conditioned supernatants of NKP_SG_ cells (green lines)_._ Control IgG1 (shadowed peaks). The data are representative of four separate experiments. The data are representative of four separate experiments. (B) RAL 555 staining shows the morphology of LPS matured myeloid DC before and after the co-culture with NKP_SG_ cells (objective x40, NA = 1). The data are representative of four separate experiments. mDC exhibit a dendritic morphology underlined by the presence of typical vacuoles and fillopodial extensions whereas causes the rapid reshaping represented by the appearance of healthy cells displaying the morphology of immature DC. (C) Nuclear localization of phospho-STAT3 (nuclear yellow staining) in six-days-old mDC before and after 1 hour of contact with NKP_SG_ cells was analyzed by confocal microscopy. DC were fixed, permeabilized, and stained for phopho-STAT3 transcription factor (green). Nuclei were stained by propidium iodide (red). As negative controls, cells were incubated with rabbit IgG, the second reagent, and propidium iodide. The data are representative of three separate experiments. (D) Flow cytometric analysis of the proliferation potential in naïve allogeneic PBMC following CFSE staining. The black columns represent the percentage of proliferating cells. The data are representative of three separate experiments. (E) Flow cytometric analysis of HLA-G molecules (continuous black line) on PB-derived monocytes treated or not with supernatant of NKP_SG_ cells. Isotype control (shadowed peak). The data are representative of three separate experiments.

Although NKP_SG cells_ expressed activating NK receptors and cytolytic granules they did not mediate natural cytotoxicity (lysis of K562 cells, Fig. 3D), failed to express the degranulation-marker CD107a and to produce IFN-γ upon cross-linking of CD16 or Natural Cytotoxicity Receptor (NCR) molecules (data not shown). The absence of the above mentioned NK cell functions led us to investigate whether NKP_SG_ could exert some unusual functions. Recent interest has focused on the interaction between NK cells and dendritic cells (DC). *In vitro* and *in vivo* studies have demonstrated various effects resulting from NK-DC interactions, including cytokine production, DC maturation and NK cell activation and proliferation [30]. Thus, we analyzed the effect of the interaction between NKP_SG_ cells and myeloid DC. CD14^+^ monocytes were cultured for 6 days in the presence of GM-CSF and IL-4 achieving their differentiation into immature dendritic cells (iDC) (CD1a^+^ CD14^−^ CD80^+^ CD83^−^ CD86^+^ HLA-DR^−^, data not shown). Their stimulation with LPS (1 µg/ml) induces within 48 hours the phenotype (CD80^++^ CD83^+^ CD86^++^ HLA-DR^high^ CD25^+^) (Fig. 6A), the morphology (development of several fillopodial extensions) (Fig. 6B) and function (IL-12 p70 production) of mature dendritic cells (mDC). Mature DC, co-cultured with NKP_SG_ cells, displayed after 48 hours a strong down-regulation of the co-stimulatory molecules (CD80 and CD86) and other maturation markers (CD83, HLA-DR and CD25). Moreover, they stopped producing IL-12p70, thus re-establishing the phenotype (Fig. 6A) and morphology of iDC (Fig. 6B). In addition, DC acquired a tolerogenic-like phenotype characterized by the induction of a membrane-bound form of TGFβ (Fig. 6A), by the nuclear translocation of pSTAT3 (Fig. 6C). Similar results were obtained in independent experiments using cell supernatants of NKP_SG_ cells. Indeed, figure 5A shows that the mDC treatment with the cell supernatants from NKP_SG_ cultures resulted in a significant decrease of the expression of CD80, CD83, CD86, HLA-DR and IL-12p70. Use of cell supernatant from conventional PB-derived NK cells had no effect (data not shown). While mDC were capable of triggering proliferation of allogeneic naïve PBMC, mDC exposed to NKP_SG_ cells appeared to be very poor inducers (Fig. 6D). After five days in culture, naïve allogeneic PBMC showed less than 10% of proliferating cells, while in sister cultures co-cultured with mitomycin-treated mDC the percentage of proliferating cells raised up to 60%. In contrast, in PBMC co-cultured with mitomycin-treated mDC previously exposed to NKP_SG_ cells only 20% underwent proliferation.

### NKP_SG_ cells induce HLA-G expression on PB monocytes and inhibit the CD107a and IFNγ surface expression on NK cells activated by NCR cross-linking

PB-derived monocytes are important effector cells of the immune response causing its activation or its down-regulation depending on the panel of the immuno-regulatory factors that they produce [31]. Thus we tested if NKP_SG_ cells could influence the behavior of PB monocytes. In figure 6E flow cytometry shows that control PB monocytes do not express on their membrane detectable amounts of HLA-G molecule, whereas 48 hours treatment with the supernatant of NKP_SG_ cells induces HLA-G molecule expression on PB monocytes.

**Figure 7:**
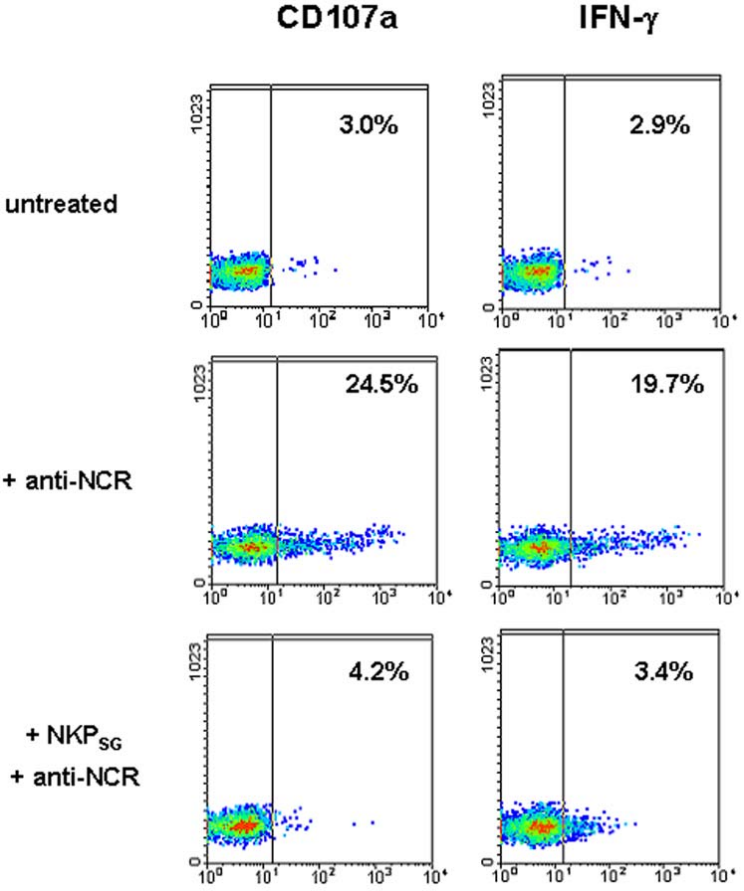
NKP_SG_ cells interfere on the lytic functions of NK cells. Dot plot analysis shows CD107a and IFNγ surface expression in conventional IL-2 activated NK cells triggered (+ anti-NCR) or not (untreated) by NCR cross-linking and after 24 hours pre-incubation with NKP_SG_ cells. In order to distinguish co-cultured NK cells from NKP_SG_ cells, these latter were stained with CFSE and excluded from cytometric analysis. The data are representative of three separate experiments.

Flow cytometric analysis (Fig. 7) showed that about 20% of human NK cells stimulated by the cross-linking of NCR expressed on their surface CD107a within 3 hours, and IFN-γ within 24 hours. When conventional PB-derived NK cells were incubated overnight with NKP_SG_ cells, a complete inhibition of CD107a expression and IFN-γ production could be detected. Remarkably, the cell supernatants obtained from NKP_SG_ cells had similar effect (data not shown).

### NKP_SG_ cells secrete tolerogenic factors

**Figure 8:**
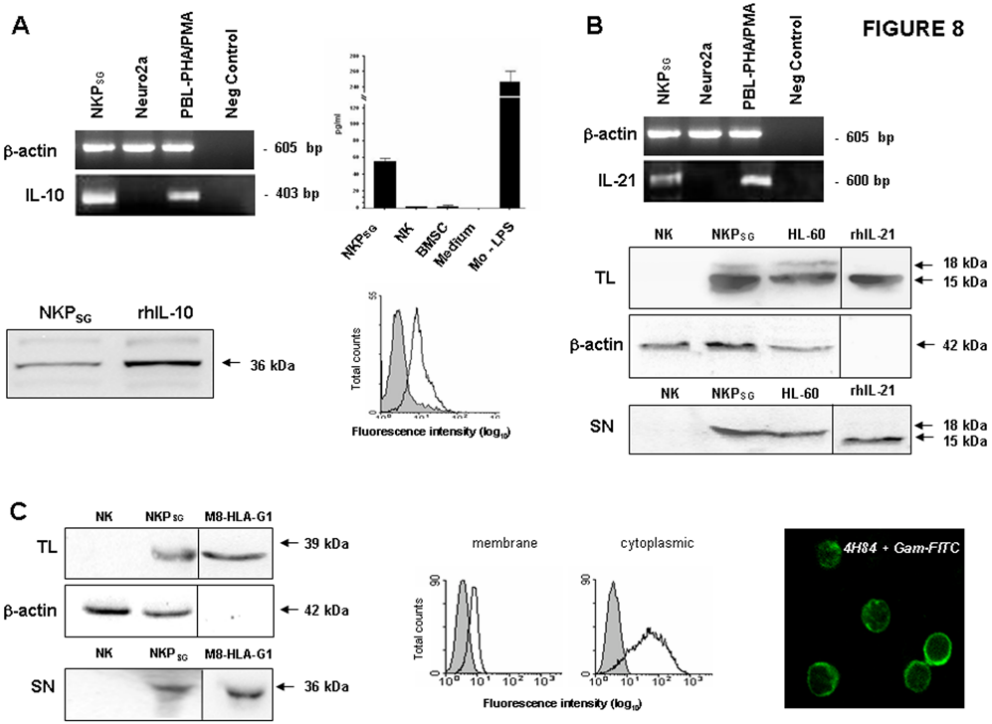
NKP_SG_ cells secrete immuno-regulatory factors. (A) Detection of IL-10 production by three-weeks-old NKP_SG_ cells using : RT-PCR analysis. The human neuroblastoma cell line Neuro2a was used as negative control, while human PBL activated with PHA and PMA were used as positive control. Amplification of the same cDNAs with β-actin-specific primers is also shown. ELISA assay. Conventional PB-NK cells and BM-MSC were used as negative controls while LPS- activated PB-macrophages were used as positive control. Western blot. recombinant IL-10 was used as positive control. Flow cytometry. Detection of membrane bound IL-10 is represented with continuous black line. Isotype control (shadowed peak). (B) Detection of IL-21 expression on three-weeks-old NKP_SG_ cells using: RT-PCR analysis. The human neuroblastoma cell line Neuro2a was used as negative control, while human PBL activated with PHA and PMA were used as positive control. Amplification of the same cDNAs with β-actin-specific primers is also shown. Western blot. IL-21 expression was analyzed in total cell lysates (TL) and cell supernantants (SN) from NKP_SG_ cells using mAbs specific for IL-21. HL-60 cell line and recombinant IL-21 were used as positive controls. Conventional PB-NK cells were used as negative control. The data are representative of three separate experiments. (C) Detection of HLA-G expression on three-weeks-old NKP_SG_ cells using: Western blot. HLA-G expression was analyzed in total cell lysates (TL) and cell supernantants (SN) from NKP_SG_ cells using mAbs specific for HLA-G. M8-HLA-G1 cell line was used as positive control. Conventional PB-NK cells were used as negative control. The data are representative of three separate experiments. Flow cytometry. Detection of surface and intracellular expression of HLA-G on NKP_SG_ cells is represented with continuous black line. Isotype control (shadowed peaks). The data are representative of three separate experiments. Confocal microscopy. As negative controls, NKP_SG_ cells were incubated with Isotype-matched IgG1-FITC.

Since the supernatant of NKP_SG_ cells displayed immuno-regulatory properties, we attempted to identify factors produced by NKP_SG_ cells that could exert tolerogenic/immuno-suppressive effects. RT-PCR analysis (Fig. 8A) revealed that NKP_SG_ cells expressed the specific transcripts for IL-10 (403 bp). Production of IL-10 was confirmed by the detection of discrete concentrations of IL-10 (30–40 pg/ml) in the cell supernatants of NKP_SG_ cells by ELISA (Fig. 8A). As negative control, we used primary cultures of human BM-derived mesenchymal stem cells [32] and IL-2 stimulated conventional NK cells, while human PB-derived monocytes stimulated with LPS were used as positive controls. In addition, Western blot analysis shows the presence of the specific 36 kDa band (Fig. 8A), and flow cytometry analysis shows that more than 80% of NKP_SG_ cells express IL-10 as a membrane-associated form, similarly to what previously reported in human monocytes releasing IL-10 [23] (Fig. 8A).

Besides IL-10 [33], another cytokine displaying similar tolerogenic effects on DC is IL-21 [34]. Thus, we investigated the production of this latter cytokine by NKP_SG_. RT-PCR analysis (Fig. 8B) revealed that NKP_SG_ cells expressed the specific transcripts for IL-21 (600 bp). Western blot analysis confirmed the presence of IL-21 (Fig. 8B) in both total cell lysates (TL) and cell supernatants (SN) from NKP_SG_ cells and of HL-60 cells used as positive control. Indeed, two specific bands of 15 and 18 kDa representing the non-glycosylated and glycosylated forms were detected in the TL of both cell types, whereas only the soluble glycosylated form (18 kDa) was detected in their SN. Resting NK cells showed no specific bands for IL-21.

IL-10 and IL-21 may account for the tolerogenic effects [33], [34] but they cannot explain the interference with the activation of NK-mediated lysis [35], [36]. Thus, we investigated whether NKP_SG_ cells could produce immuno-suppressive factors. Western blot analysis (Fig. 8C) detected a specific band of 39 kDa which identifies the membrane-bound form of HLA-G in TL from three-weeks-old NKP_SG_ cells and the M8-HLA-G1 tumor cell line used as positive control [37]. IL-2-primed NK cells were used as negative control. Moreover, Western blot analysis showed the presence of the cleaved secreted isoform of HLA-G (36 kDa) in NKP_SG_ and the control M8-HLA-G1 cell supernatants. No 36 kDa band was detected in the cell supernatants of IL-2-activated NK cells used as negative controls. Finally, intracellular and membrane expression of HLA-G by NKP_SG_ was confirmed by flow cytometry (Fig 8C) and by confocal microscopy (Fig. 8C).

### IL-10, IL-21 and soluble HLA-G modulate the immuno-regulatory properties of NKP_SG_ cells

**Figure 9:**
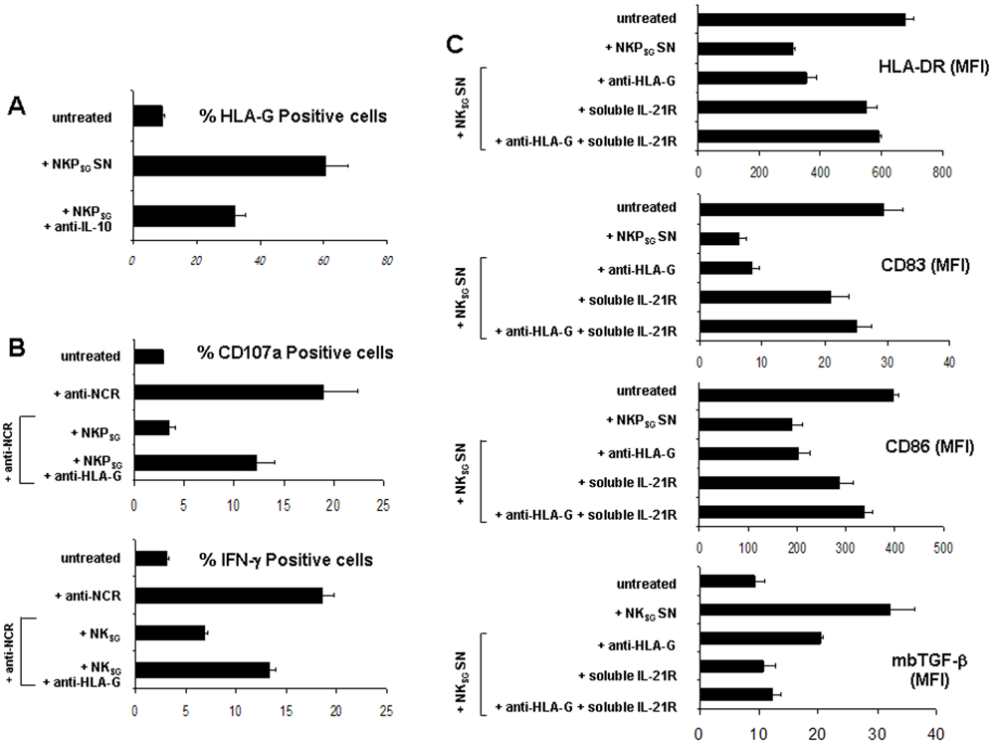
IL-10, IL-21 and HLA-G secreted by NKP_SG_ cells act as immuno-regulatory factors. (A) Effect of incubation with neutralizing anti-IL-10 mAbs (10 µg/ml) on the percentage of HLA-G positive human PB monocytes treated or not (untreated) with the cell supernantants from NKP_SG_ cells. (B) Effects of the incubation with neutralizing anti-HLA-G mAbs (10 µg/ml) on the percentage of CD107a and IFN-γ on PB-derived NK positive cells triggered with NCR cross-linking in the presence or not of NKP_SG_ cells. (C) Effects of the incubation with neutralizing anti-HLA-G mAb and/or soluble IL-21R (10 µg/ml) on the expression intensity (Mean Fluorescence Intensity) of HLA-DR, CD83, CD86 and mb-TGF-β on LPS-activated DC cultured or not with cell supernantants from NKP_SG_ cells.

The secretion by NKP_SG_ cells of potentially tolerogenic/immunosuppressive factors led us to investigate the real involvement of IL-10, IL-21 and soluble HLA-G in the immuno-regulatory properties of these cells. Analysis of figure 9A shows that less than 10% of PB-derived monocytes express on their membrane HLA-G. Incubation of these cells with the cell supernatants of NKP_SG_ cells induces within 48 hours a strong increase in the percentage of monocytes expressing HLA-G (70%). In contrast, the addition of neutralizing anti-IL-10 mAbs efficiently counteracts the stimulatory effect of the cell supernatants of NKP_SG_ cells_,_ decreasing to 35% the percentage of monocytes positive for HLA-G membrane expression.

Analysis of figure 9B shows that neutralizing anti-HLA-G mAbs efficiently counteract the inhibitory effects of NKP_SG_ cells on the membrane expression of CD107a and IFN-γ induced on PB-derived NK cells by the NCR cross-linking. In contrast, neutralization of IL-10 with specific mAbs or of IL-21 with the recombinant IL-21R/Fc chimera (s-IL-21R) had no effect (data not shown).

Analysis of figure 9C shows that the treatment of myeloid mDC with the supernatant of NKP_SG_ cells strongly decreases within 48 hours the percentage of DC expressing HLA-DR, CD86 and CD83, while it induces discrete levels of membrane-bound TGF-β. In contrast, the combined neutralization of IL-21 (s-IL-21R) and HLA-G (neutralizing mAbs) counteracts, much more efficiently than the single treatments, the suppressive effects of the cell supernatants re-establishing the expression of the above-mentioned markers.

### In vivo detection of NKp46^+^ HLA-G^+^ IL-10^+^ NK cells

**Figure 10:**
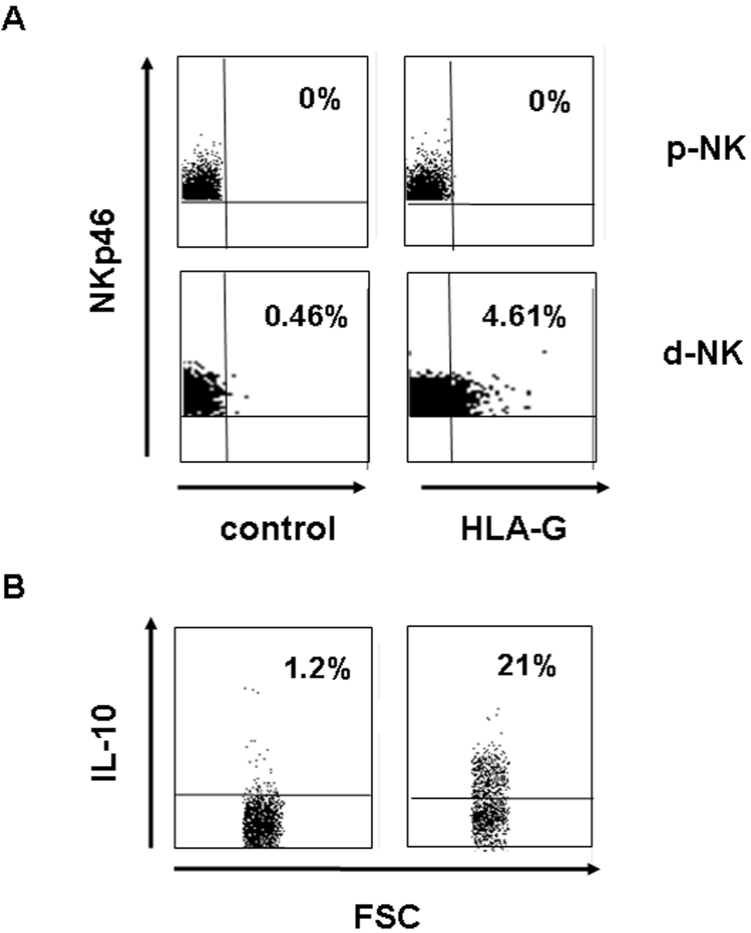
*In vivo* detection in freshly isolated decidual NK cells of a NKp46^+^ HLA-G^+^ IL-10^+^ subset. (A) Dot plot analysis of expression of NKp46 and HLA-G (surface labeling) in human freshly isolated decidual (d-NK) and PB-derived NK cells from pregnant woman (p-NK). Data are representative of six independent experiments. (B) Expression of IL-10 (internal labeling) was analyzed on the gated positive NKp46^+^ HLA-G^+^ NK cell subset.

Finally, we investigated the existence *in vivo* of NK cell subsets presenting characteristic similar to those of NKP_SG_ cells that we generate *in vitro*. Thus, we initially focused our study on the pregnancy, a physiological situation where a subset of NK cells, homing in the decidua (d-NK), spontaneously secrete IL-10 [38]. These d-NK display powerful regulatory properties, and low cytolytic activity [39] participating in the development of foetomaternal tolerance [40]. The main functional hallmark of NKP_SG_ cells is the surface expression/release of the HLA-G molecule, on the other hand, both membrane-bound and soluble HLA-G isoforms are expressed in the placenta throughout gestation playing major immunological functions and possibly acting as regulators of chorionic villous angiogenesis [41]. Therefore, we tried to identify, within the d-NK cells, a subset co-expressing NKp46, which at present is considered the most reliable marker for NK cell identification, and HLA-G. In figure 10, Dot plot analysis shows that freshly purified d-NK cells constantly express a small subset of NKp46^+^ HLA-G^+^ cells which represent about 3% of the total d-NK population. In addition, this NKp46^+^ HLA-G^+^ subset produce IL-10. In contrast this subset is not detected in the peripheral blood of pregnant women.

## Discussion

*Trans*-presentation of membrane-bound IL-15 (mb-IL-15) by BM-derived myeloid accessory cells to bystander lymphoid cells is thought to represent an important mechanism to ensure the homeostasis of CD8, NKT and NK cells [13], [14], [42]-[44]. It is likely that similar interactions may also play a major role in the development of NK cells. However, it was unclear whether IL-15 production by bystander CD34^+^ HP was required for their differentiation into NK cells [14], [16]. In this study, we provide experimental evidence that human PB-HP, but not BM-HP, constitutively co-express two types of mb-IL-15. The first one represents about 20% of the mb-IL-15 and is anchored to the cell surface through the IL-15Rα chain. The second one, which is predominant, likely represents a *trans*-membrane form as shown by the induction of reverse signaling in response to a soluble ligand and by its resistance to treatment with acidic buffer [10]. The mb-IL-15 present on these cells could mimic the effect exerted *in vivo* by mb-IL-15 expressed on BM myeloid accessory cells [13], [14] and promote *in vitro* (by reciprocal *trans*-presentation) the commitment of purified circulating progenitors towards the NK cell-lineage, in the absence of exogenous lymphokines.

Our data strongly support this hypothesis since freshly isolated CD34^+^ CD38^+^ PB-HP expanded in high density culture conditions with STEMα A medium supplemented with SCF and FL, rapidly acquire different NK cell markers (CD56, CD16, CD161, CD94) while the expression of CD34 progressively declines. Treatment with anti-IL-15Rα mAb likely interferes with the mb-IL-15 *trans*-presentation process resulting in inhibition of the NK markers and preservation of CD34 surface expression, showing that, in the absence of any detectable IL-15 secretion, the endogenous mb-IL-15 controls this process. After 3 weeks in culture, cells displayed the surface phenotype of mature activated NK cells (CD56^+^ CD16^+^ CD161^+^ CD94^+^ CD69^+^ NKG2D^+^ NKp30^+^ NKp44^+^ NKp46^+^ CCR7^+^). The activated state is revealed by the acquisition of the NCR NKp44 and of the early activation molecule CD69. The acquisition of the NKp46 receptor, the most reliable marker for NK cell identification [45], [46], indicates that these cells are truly differentiated NK cells with the potential to migrate to secondary lymphoid compartments as suggested by the expression of CCR7 [3].

Notably, they displayed a peculiar characteristic represented by the nuclear localization of the Th2 transcription factors pSTAT6 and GATA3 (NKP_SG_ cells) and by the production of the Th2 cytokines IL-4 and IL-5. The activation of the Th2 transcription factors and the production of IL-4 and IL-5 seem to be directly controlled by the mb-IL-15, as shown by their inhibition obtained incubating PB-NKP with the soluble IL-15Rα chain. The soluble ligand likely acts through an agonistic stimulation of mb-IL-15 activating a reverse signaling mechanism, even though we cannot exclude that the soluble IL-15Rα may simply behave as an antagonist interfering on the mb-IL-15 mediated *trans-*presentation process.

Interestingly, these NKP_SG_ cells did not display cytolytic functions neither against K562 cells nor after mAb-mediated triggering of CD16 and NCR receptors. We further investigated their potential to interact with DC. Various evidences of the reciprocal interactions between NK and DC have been accumulated in recent years. The potent cross-talk between these cells may lead to NK cell activation, DC activation or apoptosis, depending on the activation status of the cells, with important functional consequences on both innate and adaptive immune responses [30]. Our results highlight new outcomes of NK-DC cross-talk suggesting that NKP_SG_ cells could act as a specialized subset able to negatively shape DC maturation inducing the generation of immature/tolerogenic myeloid DC. Indeed, NKP_SG_ cells and/or their conditioned supernatants rapidly induced in mDC the down-regulation of HLA-DR, CD25, CD83, the co-stimulatory molecules CD80 and CD86. In addition, they lost the ability to produce IL-12 and to induce naive T cell proliferation. Finally, we observed the induction of important functional markers associated to the tolerogenic function such as the nuclear localization of pSTAT3 [33] and the expression of membrane-bound TGF-β [21], [47] as well as a reversion to an immature DC morphology. These data strongly support the concept that NKP_SG_ cells may display powerful regulatory properties causing the conversion of mDC into immature/tolerogenic APC. Indeed, whether DC are stimulatory or tolerogenic apparently rests with whether or not the DC express activated STAT3 [33]. On the other hand, expression of membrane-bound TGF-β on DC or Treg is responsible for the inhibition of effector functions of T and NK cells [48], [49]. In this context, it is interesting to underline that the supernatant of NKP_SG_ cells induces the membrane expression of HLA-G on human PB-derived monocytes that among circulatory mononuclear cells appear to be the predominant physiological source of these immunosuppressive molecules [37], [50]. Thus, the induction of HLA-G expression on monocytes by NKP_SG_ cell supernatants strengthens the potential role of this NK subset in down-regulation of the immune response. On the other hand, NKP_SG_ cells exhibited other important regulatory functions as they could inhibit the acquisition of cytotoxicity-associated functions (CD107a surface expression and IFN-γ production) in NK cells activated by cross-linking of NCR molecules. Since the supernatant of NKP_SG_ cells at different times of their maturation process (2 and 3 weeks-old cultures) could, at least in part, exert the same regulatory effects, we searched for the secretion of tolerogenic factors. In agreements with the expression of Th2 transcription factors, pSTAT6 and GATA3, we showed that NKP_SG_ cells produced IL-10 and IL-21. The ability of NK cells to secrete constitutively IL-10 has been recently reported in freshly isolated NK cells from HCV patients but not from healthy donors [51]. Remarkably, the concentrations of IL-10 detected in the supernatants of NKP_SG_ cells are in the range of those secreted by activated NK cells from HCV patients [51]. Therefore IL-10 secretion by NK-ireg cells is biologically relevant, as shown by the fact that its inhibition with specific neutralizing mAbs efficiently decreases the induction of HLA-G on monocytes. On the other hand, the detection on NKP_SG_ cells of IL-10 in a membrane-associated form strongly recalls an identical property in human monocytes and could be a new hallmark of the capacity described in human activated NK cells to act as APC [4]. In contrast, it was not known that human NK cells can secrete IL-21. Indeed, IL-21 production is considered to be restricted to different subsets of human CD4 Th cells [52] and to BCG-activated murine NKT cells [53]. Both IL-10 [33] and IL-21 [34] are known to alter DC differentiation, and could therefore interfere on the cross-talk between NK and DC and inhibit functional maturation of DC in efficient APC. Furthermore, IL-21 production could explain the rapid emergence of CD16^+^ NKP, that was reported to occur only when IL-21 was added to IL-15 during *in vitro* NK cell maturation [54].

IL-10 and IL-21 are likely involved in the tolerogenic property of NKP_SG_ cells, but cannot be responsible for the NKP_SG_ dependent inhibition of NK cell activation triggered by NCR cross-linking [35], [54]. However, our data show that NKP_SG_ cells express at their surface and secrete HLA-G molecules that have been shown to display immuno-regulatory properties interfering with DC maturation and NK activation [37]. The surface expression and the secretion of HLA-G are restricted to a very limited number of normal tissues in which HLA-G molecules display important immuno-regulatory functions [55]. The unusual expression and secretion of HLA-G by NKP_SG_ cells is demonstrated with four different approaches: WB, flow cytometry, confocal microscopy and biological assay using four different specific antibodies. The biological importance of HLA-G in the regulatory functions of NKP_SG_ cells is shown through its neutralization with specific mAbs. Indeed, this treatment efficiently counteracts the inhibitory effect exerted by NKP_SG_ cells on the surface expression of CD107a and IFNγ induced by NCR cross-linking on conventional PB-derived NK cells. On the other hand, the combined neutralization of HLA-G with a neutralizing mAb and of IL-21 with the soluble receptor s-IL-21R efficiently counteracts the potential of NKP_SG_ cells to induce immature myeloid DC, underlying the complementary tolerogenic properties of both factors.

In view of these properties, we propose the terminology “NK-ireg” to define this NKP_SG_ cells even if they do not express the transcription factor Foxp3 and they cannot inhibit CD3 dependent T cell expansion (data not shown). We propose that NK-ireg cells exert their immuno-regulatory potential acting through the relay of accessory cells such as monocytes and mature myeloid DC that acquire tolerogenic/suppressive properties after contact with NK-ireg cells.

We cannot exclude that NK-ireg cells could be simply immature NK cells that have not reached a stage in which they can secrete lytic granules and IFN-γ. Nevertheless, the fact that they cannot differentiate into conventional cytolytic NK cells following stimulation with IL-12, IL-15 and/or IL-18, associated to the secretion of immuno-regulatory factors (IL-21 and HLA-G) not yet detected in NK cells, and the expression of functional activation markers such as NCR NKp44 and CD69, would rather suggest that NK-ireg represent a novel truly differentiated NK subset with a self-sustainable phenotype generated from specific pSTAT6^+^ GATA3^+^ progenitors and a distinguishing surface expression of HLA-G molecules.

Recently, it has been reported the existence in the peripheral blood of a small subset of immature CD3^−^CD161^+^CD56^−^ NK cells secreting Th2 cytokines (NK2) [56]. However, comparison of this NK subset with NK-ireg cells reveals important differences that render unlikely any hypothesis of a kinship. Indeed in NK2 cells, the absence of CD56 expression seems to represent a characteristic associated to the final steps of their maturation, while NK-ireg cells acquire CD56 expression very early in their differentiation process. In addition, NK2 cells are very sensitive to the cytokine environment which rapidly activates their developmental progression towards the terminal differentiation. In contrast, NK-ireg cells and their progenitors are resistant to exogenous lymphokines and do not modify their phenotype and function. The existence of human NK cells displaying regulatory properties has been already hypothesized but not formally proven [5]. In the present report, we describe the generation *in vitro* of non-cytolytic regulatory NK cells whose differentiation is triggered by a membrane-bound IL-15 constitutively expressed by PB-derived HP but not by BM-derived ones.

The physiological significance of the NK-ireg would be strengthened by the detection *in vivo* of NK cells displaying similar phenotypic and functional characteristics. Therefore, we focused our investigation to situations where has been reported the presence of NK cells that display regulatory functions and spontaneously secrete IL-10. In mouse and human, precursors of NK cell lineage home to decidualizing uteri where they undergo proliferation, terminal differentiation and then death. Decidual NK (d-NK) cells are a distinct, transient, tissue-specific NK cell subset that during pregnancy increase along the first trimester, then decline and are virtually absent in late pregnancy. The d-NK cells spontaneously secrete IL-10 and are thought to contribute to placental angiogenesis and to play a significant role in the allorecognition mechanisms during pregnancy regulating the maternal tolerance at the foetomaternal interface. Our data show that freshly isolated d-NK cells express a small subset (3–4%) of NKp46^+^ HLA-G^+^ CD56^bright^ cells that could represent an *in vivo* counterpart of the NK-ireg that we have generated *in vitro.*

Use of immunosupressive drugs for the prevention of allograft rejection is associated with heavy side effects and toxicity [57]. In this context, the use of immuno-regulatory cells such as mesenchymal stem cells (MSC) is viewed as an interesting and promising alternative with decreased side effects [58]. Since the NK-ireg cells, that we generate *in vitro* from PB-HP, display immuno-regulatory properties similar to those reported in MSC [59], [60], we propose that this novel NK subset could constitute an additional autologous biotherapic tool to be used combined or in alternative to MSC. On the other hand, it must be stated that obtention and expansion of MSC requires bone marrow biopsies, while NK-ireg cells are derived from peripheral blood of the patient receiving an allograft.

In conclusion, the *in vitro* generation and expansion of NK-ireg cells could offer interesting perspectives for the development of new immuno-suppressive strategies in autoimmune diseases, transplant rejection or graft versus host disease.
